# Gaps Between Guidelines and Practice in Patients with Hypertension and Type 2 Diabetes: A Nationwide Cross-Sectional Study (SNAPSHOT–Brazil Study)

**DOI:** 10.3390/jcm15083022

**Published:** 2026-04-15

**Authors:** Fernanda Marciano Consolim Colombo, Dalton Bertolim Précoma, Fábio Eduardo Camazzola, Eduardo Abib Junior, Denise Reis Franco, Lucelia Batista Neves Cunha Magalhães, Antônio Carlos de Souza Spinelli, João Roberto Gemelli, João Lindolfo Cunha Borges, Renan Magalhães Montenegro Junior, Paulo Magno Martins Dourado, Renata Vital do Nascimento Lima, Mayara Lídia da Silva, Douglas Mesadri Gewehr, Alleh Nogueira, Estefane Theophilo de Freitas Pereira, Emilton Lima Junior

**Affiliations:** 1 Hypertension Unit, Heart Institute of Medical School, University of São Paulo, São Paulo 05403-900, Brazil; fernanda.consolim@incor.usp.br; 2 Sociedade Hospitalar Angelina Caron, Campina Grande do Sul 83430-000, Brazil; daltonprecoma@gmail.com; 3 Instituto de Pesquisas em Saúde (IPS), Fundação Universidade de Caxias do Sul (FUCS), Caxias do Sul 95070-561, Brazil; fecamaz1@ucs.br; 4 Scentryphar Pesquisa Clínica, Campinas 13026-501, Brazil; eabib@scentryphar.com; 5 CPclin-Centro de Pesquisas Clínicas, São Paulo 01228-200, Brazil; d9franco@gmail.com; 6 Faculdade Zarns, Salvador 41741-590, Brazil; lucelia.magalhaes@faculdadezarns.com.br; 7 Cardiocentro Natal, Natal 59020-300, Brazil; spinelli@cardiol.br; 8 Clínica Gemelli, Brasília 76804-094, Brazil; gemelli500@hotmail.com; 9 Centro de Pesquisa Clínica do Brasil, Brasília 71625-009, Brazil; jlborges@metabolismo.com.br; 10 Walter Cantidio University Hospital, Federal University of Ceará/Ebserh, Fortaleza 60110-110, Brazil; renanmmjr@gmail.com; 11 Pró Coração Cardiologia Preventiva, São Paulo 05021-010, Brazil; pmdourado@terra.com.br; 12 Medical Affairs Department, Servier Brazil Laboratories, Rio de Janeiro 22641-700, Brazil; renata.lima@servier.com (R.V.d.N.L.); mayara.silva@servier.com (M.L.d.S.); estefane.pereira@servier.com (E.T.d.F.P.); 13 Postgraduate Program in Internal Medicine and Health Sciences, Federal University of Paraná, Curitiba 80060-000, Brazil; douglasgewehr@gmail.com; 14 Postgraduate Program in Cardiology, Federal University of Rio Grande do Sul, Porto Alegre 90010-150, Brazil

**Keywords:** diabetes mellitus, glycemic control, essential hypertension, hypertension control, risk assessment, cholesterol, LDL, LDL-C control, medication adherence, physician perception

## Abstract

**Background/Objectives:** The guideline targets for blood pressure (BP), hemoglobin A1c (HbA1c), and low-density lipoprotein cholesterol (LDL-C) are frequently unmet, and physicians often misjudge control. This study aimed to characterize the real-world control of BP, HbA1c, and LDL-C in patients with type 2 diabetes (T2D) and hypertension, herein called cardiometabolic multimorbidity (CMM), and to compare guideline-based versus physician-perceived disease control. **Methods:** We conducted SNAPSHOT–Brazil, a nationwide, multicenter, cross-sectional study to gather real-world data on patients with CMM. The ESC guidelines defined the cardiovascular (CV) risk and control targets. **Results:** We included 451 patients with hypertension and T2D (median age 65 years; 60% female; 54% White). Most patients (98%) were on pharmacotherapy and reported high adherence (according to the Hill–Bone Medication Adherence Scale). A very high CV risk predominated (78%); 22% of the patients were at a high risk. The guideline-defined control was achieved in 27% for BP, 34% for HbA1c, 13% for LDL-C, and 6% for both BP and LDL-C; only 3% met all three targets simultaneously. The physicians accurately stratified the CV risk in 49% of patients, while 50% had their CV risk underestimated. They systematically overestimated control in 29% of cases for BP, 35% for LDL-C, and 25% for both. The sensitivity ranged from 0.88 to 0.98; the positive predictive values ranged from 0.19 to 0.48, and the positive likelihood ratios ranged from 2.16 to 3.65. **Conclusions:** The SNAPSHOT–Brazil study revealed a low attainment of BP, HbA1c, and LDL-C targets, despite the widespread pharmacotherapy and the high self-reported adherence. The physicians consistently overestimated disease control and underestimated the CV risk.

## 1. Introduction

Cardiometabolic diseases (CMDs), a constellation of interrelated conditions including hypertension, diabetes, obesity, dyslipidemia, and chronic kidney disease (CKD), and downstream manifestations such as coronary artery disease, stroke, heart failure, and others, remain the leading cause of death worldwide [1,2,3,4,5]. CMDs account for approximately 19–20 million deaths annually and more than 400 million disability-adjusted life-years [4,5]. The coexistence of two or more CMDs, herein referred to as cardiometabolic multimorbidity (CMM), has increased in parallel with population aging and shifts in lifestyle behaviors [1,2,3,6,7]. Hypertension affects an estimated 1.28 billion adults worldwide, and type 2 diabetes affects (T2D) over 530 million, with the prevalence of both conditions continuing to rise [4,5,8]. In Brazil, hypertension affected approximately 24% of adults in 2019, and T2D affected approximately 14% [9,10].

Attaining guideline-recommended targets for blood pressure (BP), hemoglobin A1c (HbA1c), and low-density lipoprotein cholesterol (LDL-C) is associated with substantial reductions in cardiovascular (CV) events [11,12,13,14,15]. Risk-based prevention enables physicians to tailor the treatment intensity by aligning the treatment targets and the expected life-years gained to the absolute CV risk, as assessed using calibrated tools such as SCORE2 (Systematic Coronary Risk Evaluation 2) and SCORE2–OP (SCORE2–Older Persons) [16,17]. Aligning risk categories with key treatment targets facilitates coordinated therapy and optimizes risk reduction when BP, HbA1c, and LDL-C are effectively controlled [16,17,18]. However, the achievement of these targets in real-world settings remains suboptimal, underscoring persistent evidence-to-practice gaps and therapeutic inertia [11,13,14,19].

Clinicians should actively pursue guideline-recommended targets rather than accept suboptimal control. Delays in treatment intensification or inadequate therapy frequently reflect physicians’ underestimation of the CV risk and their overestimation of disease control [11,12,19]. Near-target values can provide a misleading sense of security, as even short delays in initiating or intensifying therapy reduce the likelihood of achieving and sustaining recommended control [11,12,14,15]. All the comorbidities should be identified and addressed early, comprehensively, and intensively to minimize the residual risk [20,21,22]. Recognizing and overcoming therapeutic inertia is essential to improving long-term outcomes in patients with CMM and should be a priority for clinical practice and care quality improvement [12,20,21,22].

We conducted a nationwide, multicenter, cross-sectional study (SNAPSHOT–Brazil) to assess real-world patients with T2D and hypertension. The primary aim was to estimate the prevalence of guideline-directed target attainment for BP, HbA1c, and LDL-C, and to compare guideline-based with physician-perceived disease control.

## 2. Methods

### 2.1. Study Design and Setting

SNAPSHOT–Brazil is a nationwide, multicenter, cross-sectional study designed to assess real-world patients with T2D and hypertension. This study is part of a global initiative (SNAPSHOT) to evaluate real-world evidence across multiple countries and continents. We followed the Strengthening the Reporting of Observational Studies in Epidemiology (STROBE) reporting guideline (Supplementary Methods S1) [23]. The study was designed to enroll 400 patients between April and July 2023. Eleven healthcare centers (Supplementary Methods S2) across Brazilian cities participated in the study: São Paulo (three centers), Brasília (two centers), and one center each in Natal, Salvador, Caxias do Sul, Fortaleza, Campina Grande do Sul, and Campinas.

The protocol (DIM-05590-001-BRA) was approved by the ethics committee of the coordinating Academic Research Organization at the Heart Institute (InCor), Clinical Hospital, University of São Paulo Medical School (HC-FMUSP; approval number CAAE no. 66395322.9.0000.0068; approved on 16 February 2023). The study was also approved by the ethics committee of each participating center and was conducted in accordance with the principles of the Declaration of Helsinki [24].

### 2.2. Study Population

This study included adults aged 18 years or older with a confirmed diagnosis of T2D and hypertension. Hypertension was defined as an office-measured systolic blood pressure (SBP) ≥ 140 mm Hg and/or a diastolic blood pressure (DBP) ≥ 90 mm Hg, or the current use of antihypertensive medications. T2D was defined by an HbA1c level ≥ 6.5% (or ≥48 mmol/mol), or the current use of glucose-lowering medications, and was confirmed by the investigator at the time of the study visit through a rigorous retrospective review of the patient’s medical records in addition to a clinical evaluation and direct questioning, thereby excluding other forms of diabetes. Dyslipidemia was defined by an investigator-reported medical history or the current use of lipid-lowering medications. All participants provided written informed consent. The patient participation was voluntary, and no data were collected from individuals who declined.

### 2.3. Data Collection, Variables, and Measurements

The data collection for each participant occurred during a single, routine outpatient visit, ensuring that the clinical care reflected real-world practice and was not influenced by study-specific interventions or additional assessments. All evaluations and data collection were performed by board-certified specialists in cardiology or endocrinology. Each patient’s involvement was limited to the duration of that visit.

The investigators collected demographic and epidemiological data, CV risk factors, comorbidities, and details of current treatments. BP was measured according to the 2020 International Society of Hypertension guidelines, with three readings 1 to 2 min apart, and the average of the final two recorded [25]. Heart rate was measured in the seated position after 5 min of rest. The data on LDL-C, HbA1c, fasting blood glucose, current treatments, and micro- and macrovascular complications were obtained from medical records, using the most recent assessment within the past year.

The patient-reported outcomes were collected using a self-administered paper questionnaire completed independently at the end of their routine visit, ensuring no investigator influence. This questionnaire included three sections: (1) social information, encompassing education level, marital status, socio-professional category, income, and physical activity; (2) the Patient Health Questionnaire-9 (PHQ-9), a validated depression screening tool (scored 0–27, with higher levels indicating more severe depressive symptoms and a score ≥ 10 denoting a positive screen) [26,27]; and (3) the Hill–Bone Medication Adherence Scale (HB-MAS), which assessed the medication adherence to medications for hypertension, hypercholesterolemia, and T2D (scored 9–36, with higher scores reflecting better adherence) [28]. A detailed description of the questionnaire is provided in Supplementary Methods S3.

All the participant data were recorded in an electronic case report form (Supplementary Methods S4) and pseudonymized to preserve confidentiality. The data collection, including patient-reported outcomes, was conducted under the oversight of an authorized investigator. The dataset was managed by a contract research organization in accordance with standard operating procedures. The detailed variable definitions for categorical thresholds and data handling procedures are provided in Supplementary Methods S5.

### 2.4. Outcomes

We evaluated the disease control status and CV risk separately from two perspectives: guideline-directed criteria and physician-perceived assessments by cardiologists and endocrinologists. The guideline-directed control was assessed for BP, HbA1c, and LDL-C, whereas the physician-perceived control was only appraised for BP and LDL-C (as prespecified in the protocol). The control status for hypertension, T2D, and LDL-C was defined according to the 2018–2019 European Society of Cardiology (ESC) guideline recommendations, with the full criteria detailed in Supplementary Methods S5 [18,29,30]. The ESC guidelines were selected to ensure methodological consistency and international comparability, as this study is part of the SNAPSHOT global initiative. Hypertension control varied by age, treatment status, and comorbidities. In untreated patients, BP < 140/90 mm of mercury (mm Hg) (for individuals < 80 years) or <160/90 mm Hg (for those ≥80 years) was considered controlled. In treated patients, control was defined as <130/80 mm Hg (for individuals < 65 years without CKD) or <140/80 mm Hg (for those ≥65 years or with CKD). T2D control was defined as HbA1c < 7% (<53 mmol/mol), while LDL-C targets were risk-stratified according to the ESC categories (<70 mg/dL [milligrams per deciliter] for patients at a high CV risk and <55 mg/dL for those at a very high risk) [18,30].

The CV risk was assessed according to the 2019 and 2020 ESC guidelines (Supplementary Methods S5). SCORE2 and SCORE2–OP were calculated using the ‘RiskScorescvd’ R package [18,30,31]. Although Brazil is not in Europe, it was classified as a high-risk region for SCORE2 and SCORE2-OP calculations based on prior studies demonstrating that the cardiovascular risk among Brazilian individuals is comparable to that in high-risk European populations. By study design, all participants were classified as at least high CV risk. The participants were classified as very high risk if they had established ASCVD, T2D with target-organ damage or long duration (>20 years), severe CKD (estimated glomerular filtration rate < 30 mL/min/1.73 m^2^), or another major risk factor (Supplementary Methods S5). For those without such determinants, the 10-year risk was estimated using SCORE2 and SCORE2-OP [16,17].

### 2.5. Statistical Analysis

The minimum sample size was 400 patients, calculated assuming a 20% prevalence, an alpha of 0.05, and a 4% margin of error for estimating the proportion of patients who are simultaneously controlled for hypertension and T2D [32]. The descriptive analyses summarized sociodemographic variables, BP, HbA1c, LDL-C levels, CV risk, and control of hypertension, T2D, and LDL-C. The categorical variables are presented as counts and percentages, and the continuous variables are presented as mean ± standard deviation or median (interquartile range [IQR]), as appropriate. Wilson 95% confidence intervals (CI) for binomial proportions were estimated. The variables with missingness ≥ 10% and <50% were imputed. For the continuous variables, we applied median imputation to preserve central tendency and reduce the influence of skewed distributions. No categorical variables met the criteria requiring imputation. Moreover, imputation was limited to the baseline and sociodemographic variables; no outcome data were imputed.

The associations between the control status or the CV risk categories and sociodemographic or clinical characteristics were examined. We compared the prevalence of disease control based on guideline-directed thresholds versus physician-perceived assessments. For each outcome, we calculated the paired prevalence difference (physician minus guideline) with 95% CIs, and we formally tested the paired comparisons using McNemar’s test. Using the guideline-directed control as the reference, the physician-perceived control was evaluated by sensitivity, specificity, positive and negative predictive values (PPV, NPV), likelihood ratios (LR+, LR−), overall accuracy, and discrimination (area under the receiver operating characteristic [ROC] curve). The multivariable logistic regression was performed to identify independent predictors of BP, LDL-C, and HbA1c control. The candidate variables were selected a priori based on clinical plausibility and established evidence from prior studies. All analyses were conducted using R v4.2.2 (R Core Foundation) [33].

## 3. Results

### 3.1. Sociodemographic and Clinical Characteristics

A total of 451 patients with hypertension and T2D were interviewed between April and July 2023 and were included in the analysis. The median age of patients was 65 (IQR, 59–72) years, and 95% were aged 50 years or older. Overall, 60% were women, and 219 (54%) identified as White. The patients classified as very high-risk patients were more likely to be White, whereas the high-risk group included higher proportions of Black or African American and Hispanic or Latino individuals (*p* < 0.001). The anthropometric and sociodemographic characteristics are summarized in Table 1 and Table 2.

The median BMI was 30 kg/m^2^ (IQR, 26–33), and the mean waist circumference was 101 cm (IQR, 91–110). In addition, 35% of patients were overweight, and 48% were obese. The BMI was higher in the high-risk group compared with the very high-risk group (31 [IQR, 28–36] vs. 29 [IQR, 26–33]; *p* < 0.001). Overall, 7% were current smokers and 34% were former smokers, with a median cessation duration of 20 years (IQR, 11–30).

Most participants were married or in a relationship (53%) and had low educational attainment (79%). Moreover, 23% were employed, 58% reported low income, and 44% had sedentary lifestyles. Marital status differed significantly between the risk groups: high-risk patients were more often single, while those at very high risk had higher proportions of widowed individuals. Unemployment was significantly greater among very high-risk patients compared with those at a high risk (82% vs. 63%; *p* < 0.001). The median PHQ-9 score was six (IQR, 2–11), and 31% of the participants screened positive for depressive symptoms (PHQ-9 score ≥ 10).

CMM was common, with 93% of patients having at least one comorbidity in addition to hypertension and T2D (Table 3). The most frequent additional conditions were dyslipidemia (88%), chronic coronary syndrome (23%), chronic kidney disease (27%), peripheral arterial disease (24%), prior myocardial infarction (19%), and sleep disorders (13%). Evidence of target-organ damage was present in 55% of patients, including left ventricular hypertrophy in 31%.

Most patients were classified as very high risk for CV events (Table 3). According to the ESC guidelines and SCORE2/2-OP calculators, the median estimated 10-year risk was 19% (95% CI, 12–30%), with 22% (95% CI, 18–26%) at a high risk and 78% (95% CI, 74–82%) at a very high risk.

### 3.2. BP Profile and Antihypertensive Therapy

Supplementary Table S1 details the BP profile and antihypertensive therapy patterns. The median hypertension duration was 16 years (IQR, 9–25), with the longest duration observed in patients at a very high risk and with uncontrolled hypertension (*p* < 0.001 and *p* = 0.03, respectively). The median SBP and DBP were 140 mm Hg (IQR, 126–158) and 80 mm Hg (IQR, 70–90), respectively. The patients at a very high risk had a higher SBP than those at a high risk (140 mm Hg [IQR, 127–160] vs. 133 mm Hg [IQR, 122–143]; *p* = 0.001). A total of 56% of patients had SBP/DBP ≥ 130/85 mm Hg, and 27% only exhibited SBP above the recommended target.

Antihypertensive therapy was administered to 98% of the participants, with a median total daily pill burden of four tablets (IQR, 2–6). The patients at a very high risk had a significantly higher daily pill load than those at a high risk (four tablets [IQR, 2–7] vs. three tablets [IQR, 2–4]; *p* < 0.001). Single-pill combination therapy accounted for 16% of the treated individuals. Diuretics (59%), β-blockers (55%), and angiotensin receptor blockers (ARB; 51%) were the most frequently prescribed classes, followed by calcium channel blockers (CCB; 49%) and angiotensin-converting enzyme inhibitors (ACEI; 40%). The use of CCBs and β-blockers was significantly higher among patients classified as very high risk. The median HB-MAS adherence score for antihypertensive therapy was 35 (IQR, 33–36), which was consistent with the high self-reported adherence across the cohort.

### 3.3. Glycemic Profile and T2D Treatment

Supplementary Table S2 presents glycemic profiles and T2D treatment patterns. The median T2D duration was 12 years (IQR, 5–20). The mean HbA1c was 7% (IQR, 6.5–8.0%), and the median fasting glucose was 129 mg/dL (IQR, 107–161). Overall, 66% of the patients had an HbA1c ≥ 7%, and 32% had an HbA1c ≥ 8%.

Most patients (98%) were receiving glucose-lowering pharmacotherapy, with a median daily pill burden of three tablets (IQR, 2–4). Single-pill combination formulations were used by 16% of the treated individuals. Metformin was the most frequently prescribed agent (83%), followed by insulin (34%), sodium–glucose cotransporter 2 (SGLT2) inhibitors (32%), and sulfonylureas (30%); glucagon-like peptide-1 (GLP-1) receptor agonists were used in 5% of patients. Among the sulfonylurea users, second-generation agents (glibenclamide, gliclazide, and glipizide) accounted for 78%, and third-generation agents (gliclazide MR, glimepiride, and glipizide XL) accounted for 22%. The median HB-MAS score for glucose-lowering therapy was 35 (IQR, 33–36).

### 3.4. Lipid Profile and Lipid-Lowering Treatment

Supplementary Table S3 details the lipid profiles and treatment patterns. The median time since hypercholesterolemia diagnosis was 10 years (IQR, 5–16), and the median LDL-C was 85 mg/dL (IQR, 71–103). Overall, 77% had LDL-C levels ≥ 70 mg/dL, and 89% had levels ≥ 55 mg/dL.

Lipid-lowering therapy was administered to 84% of the participants, most commonly statins (83%). High-intensity statins were prescribed in 42% of cases, and ezetimibe was prescribed in 12%. The patients at a very high CV risk were more likely than those at a high risk to receive statin therapy (86% vs. 75%; *p* = 0.011), including high-intensity statins (*p* < 0.001). Single-pill combinations were used in 10% of the treated patients. The median HB-MAS score for lipid-lowering therapy was 35 (IQR, 33–36).

### 3.5. Guideline-Directed and Physician-Perceived Control

**Figure 1:**
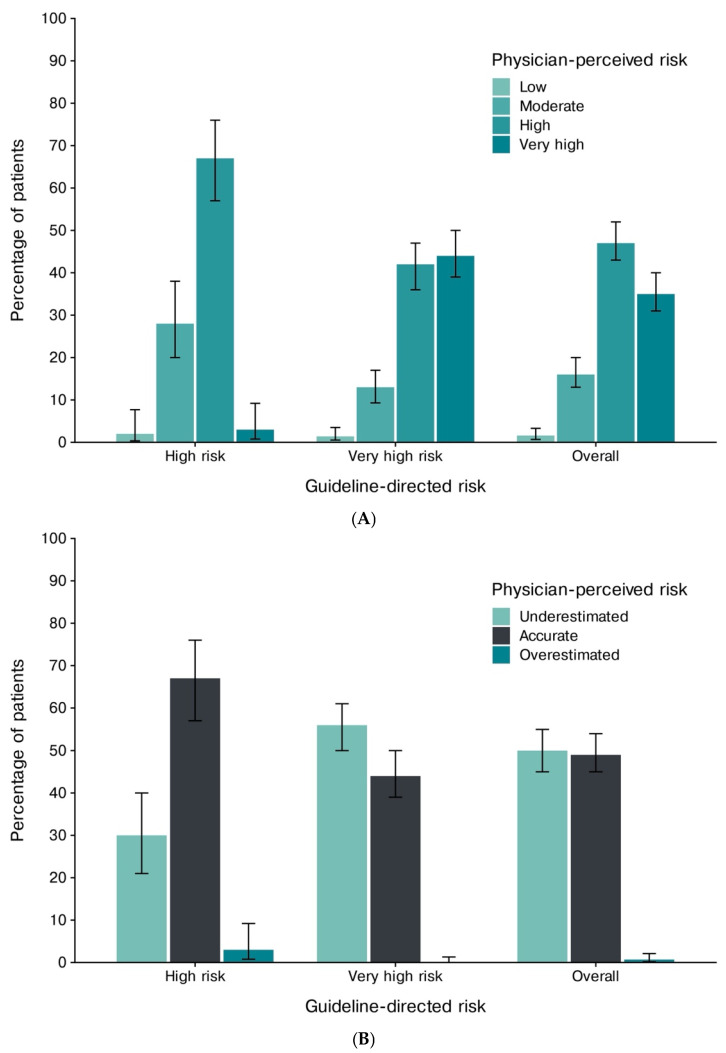
The guideline-directed versus physician-perceived cardiovascular risk, overall and by cardiovascular risk. (**A**) The distribution of physician-perceived cardiovascular risk categories (low, moderate, high, and very high) within the guideline-directed risk strata (high and very high) and overall. (**B**) The accuracy of physicians’ risk assessments (underestimation, accurate estimation, and overestimation) across the same guideline-directed strata and overall. The bars indicate the estimated proportions, with the whiskers representing the 95% confidence intervals.

On the basis of the physician assessment (Figure 1A; Supplementary Table S4), 1.6% of patients (95% CI, 0.7–3.3%) were classified as having a low CV risk, 16% (95% CI, 13–20%) as having a moderate risk, 47% (95% CI, 43–52%) as having a high risk, and 35% (95% CI, 31–40%) as having a very high risk. In contrast, the application of the ESC criteria and SCORE2/2-OP categorized no patients as having a low-to-moderate CV risk; 22% (95% CI, 18–26%) were classified as high risk, and 78% (95% CI, 74–82%) as very high risk. Overall, 49% of the participants (95% CI, 45–54%) were correctly stratified, whereas physicians underestimated the CV risk in 50% (95% CI, 45–55%); an overestimation of the risk occurred with 0.7% of the patients (Figure 1B).

**Figure 2:**
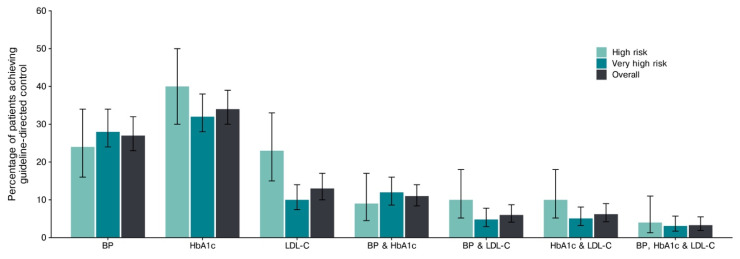
The proportion of patients achieving guideline-directed control, overall and by cardiovascular risk. The overall and cardiovascular risk-stratified proportions of patients achieving guideline-recommended control for BP, HbA1c, LDL-c, and their combined endpoints. The bars indicate the estimated proportions, with whiskers representing the 95% confidence intervals. Abbreviations: BP, blood pressure; HbA1c, hemoglobin A1c; and LDL-C, low-density lipoprotein cholesterol.

**Figure 3:**
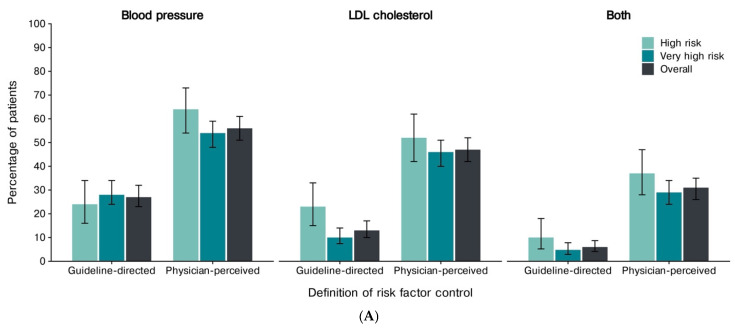

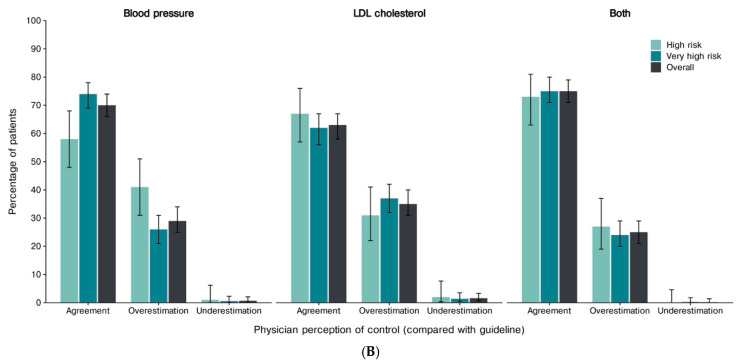
The guideline-defined vs. physician-perceived control across cardiovascular risk strata, overall and by cardiovascular risk. (**A**) The overall and cardiovascular risk-stratified proportions of patients achieving guideline-defined versus physician-perceived control for blood pressure, LDL-C, and their combined endpoint. (**B**) The accuracy of physicians’ assessments of control (underestimation, agreement, and overestimation) across the guideline-directed strata and overall is shown separately for BP, LDL-C, and both. The bars indicate the estimated proportions, with whiskers representing the 95% confidence intervals. Abbreviations: LDL-C, low-density lipoprotein cholesterol.

The guideline-defined control was achieved in 27% (95% CI, 23–32%) for BP, 34% (95% CI, 30–39%) for HbA1c, 13% (95% CI, 10–17%) for LDL-C, 6% (95% CI, 4–9%) for combined BP and LDL-C control, 11% (95% CI, 8–14%) for combined BP and HbA1c control, and 6% (95% CI, 4–9%) for combined LDL-C and HbA1c control (Figure 2; Supplementary Table S4). Notably, only 3% (95% CI, 2–6%) met all three targets (BP, LDL-C, and HbA1c) (Figure 2; Supplementary Table S4). Physicians consistently overestimated control, classifying 56%, 47%, and 31% of patients as controlled for BP, LDL-C, and combined BP and LDL-C targets, respectively (Figure 3A,B; Supplementary Table S4). This resulted in an absolute overestimation of 29% (95% CI, 22–35%; *p* < 0.001) for BP control, 35% (95% CI, 28–40%; *p* < 0.001) for LDL-C, and 25% (95% CI, 20–30%; *p* < 0.001) for both BP and LDL-C (Supplementary Table S4).

The agreement between physician-perceived and guideline-defined control was 70% (95% CI, 66–74%) for BP, 63% (95% CI, 58–67%) for LDL-C, and 75% (95% CI, 71–79%) for combined BP and LDL-C (Figure 3B; Supplementary Table S4). The physicians predominantly overestimated control (BP 29%, LDL-C 35%, combined 25%), whereas underestimation was uncommon (<2% across all measures).

### 3.6. Diagnostic Accuracy of Physician Perception

The diagnostic performance measures showed modest accuracy of the physician-perceived control across outcomes (Supplementary Table S5). The sensitivity estimates were 0.98 (95% CI, 0.93–0.99) for BP, 0.96 (95% CI, 0.81–1.00) for combined BP and LDL-C, and 0.88 (95% CI, 0.77–0.95) for LDL-C alone. The specificity ranged from 0.59 to 0.74, LR (+) ranged from 2.16 to 3.65, and LR (−) ranged from 0.04 to 0.20. The PPVs ranged from 0.19 to 0.48, whereas the NPVs ranged from 0.97 to 1.00. The areas under the ROC curve were 0.74 for LDL-C, 0.79 for BP, and 0.85 for the combined control. The overall accuracy ranged from 0.63 to 0.75

### 3.7. Multivariable Analysis

The multivariable logistic regression analysis (Supplementary Table S6) showed that older age (odds ratio [OR], 1.03 per year; 95% CI, 1.00–1.07), post-secondary education (OR 3.21; 95% CI, 1.32–7.98), and single-pill combination therapy (OR 1.97; 95% CI, 1.05–3.67) were independently associated with greater odds of BP control. Abdominal obesity (OR 0.52; 95% CI, 0.31–0.87), each additional daily pill (OR 0.90; 95% CI, 0.82–0.98), and longer disease duration (OR 0.89 per 5 years; 95% CI, 0.79–1.00) were associated with lower odds of BP control.

For HbA1c control, older age (OR 1.03 per year; 95% CI, 1.00–1.06), post-secondary education (OR 5.07; 95% CI, 1.92–14.5), and HB-MAS adherence score (OR 1.11 per point; 95% CI, 1.02–1.24) were independently associated with higher odds of achieving HbA1c control. Microvascular complications (OR 0.58; 95% CI, 0.34–0.98), each additional daily pill (OR 0.67 per pill; 95% CI, 0.56–0.80), and longer diabetes duration (OR 0.81 per 5 years; 95% CI, 0.71–0.92) were associated with lower odds of HbA1c control. Older age (OR 1.04 per year; 95% CI, 1.01–1.06) and post-secondary education (OR 4.48; 95% CI, 1.89–11.3) were also independently associated with achieving LDL-C control.

## 4. Discussion

SNAPSHOT–Brazil is a national, multicenter, cross-sectional survey designed to characterize real-world data and identify gaps in care for patients with CMM. The main findings were (Graphical Abstract): (1) the attainment of the guideline-directed targets for BP, HbA1c, and LDL-C was limited, despite high pharmacotherapy use and self-reported adherence; (2) the control rates were lower when all three targets were considered simultaneously; (3) the physicians frequently overestimated disease control and underestimated the CV risk relative to the guideline-defined thresholds, indicating a limited accuracy in their assessments; and (4) in secondary, multivariable-adjusted analyses, several factors were independently associated with target attainment, including use of single-pill combination therapy with greater odds of blood-pressure control, post-secondary education with greater odds of achieving blood pressure, LDL-C, and HbA1c targets, and higher daily pill burden with lower odds of meeting blood pressure and HbA1c goals.

The inadequate treatment of hypertension and the overestimation of BP control, particularly among patients at high and very high CV risk, contribute to the progression of target-organ damage and an increased risk of CV events. The evidence from randomized trials, including SPRINT, STEP, BPROAD, and ESPRIT, indicates that intensive BP control substantially reduces the CV risk, highlighting the need for accurate BP assessment and timely treatment intensification [34,35,36,37]. Despite the strong supportive evidence, the real-world achievement of recommended targets remains disappointing, reflecting persistent evidence-to-practice gaps. A pooled analysis by the NCD Risk Factor Collaboration, encompassing 1201 population-representative studies with 104 million participants, reported that only 18–23% met the guideline-recommended targets [38]. In a European real-world survey, 40 to 60% of patients did not reach the target levels despite adequate adherence, and fewer than 10% of those with T2D achieved BP control [39]. Our study indicates that limited target attainment may partly stem from the systematic underestimation of CV risk and overestimation of cardiometabolic control in routine practice. Importantly, BP targets were defined according to the 2018–2019 ESC guidelines that were applicable at the time of data collection [29]. Application of the lower targets proposed in the 2024 ESC guidelines should be the preferred approach in future studies and would likely result in even lower BP control rates, further underscoring the magnitude of the observed care gaps [22].

The Brazilian data provide a somewhat more favorable but still suboptimal picture. The 2013 Brazilian National Health Survey reported that 51.4% (95% CI, 45.7–57.1%) of patients with T2D achieved the BP goals, whereas only 28.4% (95% CI, 17.2–39.5%) did among those at a high CV risk [32]. Similarly, the Brazilian registry of atherothrombotic disease (NEAT study) found that 40.7% (95% CI, 38.6–42.9%) of patients with coronary and/or peripheral arterial disease attained the BP targets [40]. In our survey of high- and very-high-risk patients receiving specialist care, fewer than one-third (27%; 95% CI, 23–32%) achieved the BP targets despite a high reported adherence, and 29% of patients with objectively uncontrolled hypertension were perceived as controlled. Our study underscores the frequent overestimation of BP control in routine practice, which is a challenge that is documented across diverse clinical settings in previous studies [41,42,43,44].

Although all the patients in our survey were, by definition, at least at a high CV risk, based on the inclusion criteria of hypertension and T2D, 18% of the investigators classified them as having low-to-moderate risk. Notably, all the participating investigators were board-certified cardiologists or endocrinologists. Overall, 49% of the patients were correctly risk-stratified, while 50% had their CV risk underestimated. These results are consistent with prior studies showing physicians frequently misclassifying CV risk, even when the guidelines’ definitions are explicit [45,46,47].

In the GOULD Registry, a US prospective cohort of over 5000 patients with ASCVD, only 17% received high-intensity statins and fewer than 25% reached LDL-C < 70 mg/dL [45]. A multicenter cross-sectional study from Spain found that physicians overestimated LDL-C control in patients with dyslipidemia (62% perceived vs. 31% actual), with the discrepancy greater among those at a high and a very-high CV risk [46]. Similar findings were reported in multicenter studies from Italy and Malaysia, in which physician-perceived risk underestimated the actual CV risk in 37% and 60% of patients, respectively [47,48]. These results highlight a notable discrepancy between the physician-perceived risk and the guideline-derived risk assessments. Our results continue to raise a critical concern; if specialists underestimate the CV risk, misclassification is likely even greater in primary care, where clinicians manage the majority of patients with CMD under time- and resource-constrained conditions. Limited opportunities for comprehensive risk assessment in this setting may result in patients who would benefit from intensive preventive interventions being overlooked, perpetuating gaps in risk control and missed opportunities to prevent adverse CV outcomes.

In our survey, 34% of patients (95% CI, 30–39%) achieved glycemic control (HbA1c < 7%), lower than what was reported in US NHANES (50.5%) and Korean NHANES (52.6%) [49,50]. Similarly, the 2013 Brazilian National Health Survey found that 46% (95% CI, 40–52%) of patients did not reach HbA1c targets [32]. In the NEAT study, which included patients at a very high cardiovascular risk, glycemic control was even poorer, with only 15.7 (95% CI, 13.5–18.3%) achieving HbA1c targets [40]. The control of LDL-C was also suboptimal, with 31% of patients reaching guideline-recommended goals in a multicenter Spanish survey, 40% (95% CI, 35–46%) in the 2013 Brazilian National Health Survey, and 8.6% (95% CI, 7.4–9.9%) in the NEAT study (LDL-c goal of <55 mg/dL) [32,46].

By considering all three targets, BP, HbA1c, and LDL-C, we have widened the evidence-to-practice gap. In the US NHANES, 22.2% of people met all three criteria (A1C < 7%, blood pressure < 140/90 mm Hg, and non-HDL cholesterol < 130 mg/dL) [50]. In contrast, the Korean NHANES, which applied more stringent targets (A1C < 6.5%, blood pressure < 140/85 mm Hg, and LDL-C < 100 mg/dL), reported that only 8.4% achieved all three goals [49]. Similarly, the 2013 Brazilian National Health Survey found that only 12.4% reached control across all three parameters [32]. In our survey, the proportion was markedly lower, with only 3% (95% CI, 2–6%) of patients meeting all three targets.

Several reasons may contribute to these findings. First, despite the proven efficacy of the available therapies, physicians often rely on monotherapy or suboptimal medication doses, contrary to guideline recommendations. Second, insufficient therapy, which may result from pill burden, perceived drug adverse effects, or other barriers. In our study, higher self-reported adherence was independently associated with HbA1c control, whereas greater pill burden was associated with lower odds of achieving BP and HbA1c targets. Third, the limited consultation time and rising clinical demand mean short visits, heavy caseloads, and minimal support can hinder comprehensive evaluation and the use of formal risk-assessment tools, leading to rapid, imprecise clinical judgments and downstream missed treatment opportunities. Fourth, limited patient education and understanding of individual cardiovascular risk may reduce engagement, adherence, and shared decision-making. Fifth, a lack of familiarity with contemporary risk-stratification algorithms reduces the likelihood that physicians routinely apply SCORE2/2-OP or similar tools in practice. Finally, the multivariable analyses showed that single-pill combination therapy was independently associated with BP control, and higher patient education (post-secondary schooling) was independently associated with achieving the BP, HbA1c, and LDL-C targets. These findings underscore the potential benefits of simplified treatment regimens, reduced pill burden, patient-level education, and electronic health record-based decision support (including automated risk calculation) in improving cardiometabolic risk-factor management.

Our findings highlight the urgent need for a truly comprehensive management of CMM. *“Almost in target is out of target”* underscores that the near-optimal control of blood pressure, glycemia, or LDL-C is insufficient to reduce risk in patients with hypertension and diabetes meaningfully. *“The invisible patient”* reflects the persistent residual risk driven by unrecognized comorbidities, missed diagnoses, and incomplete risk assessment. Ultimately, a *“no one left behind”* approach is imperative and is one that identifies, addresses, and aggressively treats every modifiable risk factor. Only consistent, guideline-based, multifactorial care can close the gaps revealed by our survey.

### Limitations

This study has limitations. First, the cross-sectional, observational design with a single routine-care visit precludes temporal inference. It is susceptible to measurement variability, including the white-coat effect and regression to the mean. In addition, the BP assessment was based on office measurements without the systematic use of out-of-office monitoring, such as home BP monitoring or 24 h ambulatory blood pressure monitoring, which may have led to the misclassification of BP control. Second, we did not apply the SCORE 2-Diabetes algorithm because the estimated glomerular filtration rate (eGFR) or albuminuria (e.g., urinary albumin-to-creatinine ratio) were unavailable. Thus, chronic kidney disease was assessed in a binary manner. Third, the treatment patterns were described without detailed clinical indication. Fourth, the key variables relevant to contemporary cardiometabolic management were not available, including advanced lipid markers (e.g., apolipoprotein B, non-HDL cholesterol, and lipoprotein(a)). Fifth, the sleep disorders were self-reported without standardized screening for obstructive sleep apnea, which may have led to an underestimation of an important contributor to resistant hypertension. Sixth, in multivariable-adjusted analyses, several clinically plausible factors may not have reached statistical significance because of limited power and should therefore be interpreted in the context of their rationale, direction of association, effect size, and confidence interval precision rather than their *p*-values alone. Seventh, only patients attending routine medical appointments were included, which may have led to an overestimation of treatment adherence and cardiometabolic target attainment, as individuals who were not engaged in regular follow-ups tended to have poorer BP, HbA1c, and lipid control. Eight, the applicability may be limited to healthcare systems with organizational structures and care pathways similar to those in Brazil. Finally, the study was supported by industry funding, which may raise concerns regarding the potential sponsor-related bias, particularly in the context of the treatment strategies discussed. Although the study was designed and conducted independently, this aspect should be considered when interpreting the findings.

## 5. Conclusions

The SNAPSHOT–Brazil cross-sectional study reveals low attainment of BP, HbA1c, and LDL-C targets among patients with CMM, despite widespread pharmacotherapy use and self-reported adherence. The physicians’ perceptions significantly overestimated the disease control and underestimated the CV risk relative to the objective guideline thresholds, highlighting a systematic misclassification and suboptimal accuracy in routine practice. Given that this study was conducted in specialized care settings, these findings likely represent a conservative estimate, and the magnitude of care gaps may be even greater in primary care.

## Figures and Tables

**Table 1:** The sociodemographic characteristics of the study population, overall and stratified by cardiovascular risk according to guideline recommendations.

	Overall (*n* = 451)	Cardiovascular Risk		*p*-Value ^1^
		High Risk *n* = 100	Very High Risk *n* = 351	
Age, years	65 [59–72]	59 [55–63]	68 [63–73]	<0.001
Female gender	269 (60)	68 (68)	201 (57)	0.054
Race				<0.001
White	244 (54)	37 (37)	207 (59)	
Hispanic or Latino	105 (23)	31 (31)	74 (21)	
Black or African	99 (22)	32 (32)	67 (19)	
Asian	3 (0.7)	0 (0)	3 (0.9)	
BMI, kg/m^2^	30 [26–33]	31 [28–36]	29 [26–33]	<0.001
25–29.9 kg/m^2^	157 (35)	29 (29)	128 (36)	
≥30 kg/m^2^	217 (48)	65 (65)	152 (43)	
Waist size, cm	101 [92–110]	103 [97–110]	100 [92–110]	0.11
Smoking habit				0.10
Smoker	31 (7)	5 (5)	26 (7)	
Non-smoker	265 (59)	68 (68)	197 (56)	
Former smoker	155 (34)	27 (27)	128 (36)	
Time since smoking cessation, years	20 [11–30]	23 [14–30]	20 [10–30]	0.3
Marital status				0.001
Single	68 (15)	24 (24)	44 (13)	
Married or in a relationship	240 (53)	53 (53)	187 (53)	
Divorced	63 (14)	16 (16)	47 (13)	
Widowed	80 (18)	7 (7)	73 (21)	
Educational level	n = 450		n = 350	--
Low education *	356 (79)	68 (68)	288 (82)	
Medium education †	64 (14)	18 (18)	46 (13)	
High education ‡	30 (7)	14 (14)	16 (5)	
Currently employed	103 (23)	37 (37)	66 (19)	<0.001
Self-reported low income	260 (58)	51 (51)	209 (60)	0.13
Sedentary lifestyle	200 (44)	44 (44)	156 (44)	>0.9
PHQ-9 score	6 [2–11]	6 [2–12]	7 [3–10]	0.9
Positive depression screen (PHQ-9 ≥ 10)	138 (31)	33 (33)	105 (30)	0.6

Caption: The data are *n* (%) and median [IQR]. Abbreviations: BMI, body mass index; cm, centimeters; kg, kilograms; m, meters; SD, standard deviation; T2D, type 2 diabetes. ^1^ the Wilcoxon rank sum test for continuous variables; Pearson’s Chi-squared or Fisher’s exact tests for categorical variables. * less than primary, primary, and lower secondary. † upper secondary and post-secondary non-tertiary. ‡ short-cycle tertiary, bachelor’s or equivalent, master’s, and doctoral education.

**Table 2:** The sociodemographic characteristics of the study population stratified by disease control status according to guideline recommendations.

	Overall (*n* = 451)	Hypertension (*n* = 451)		*p*-Value ^1^	T2D (*n* = 451)		*p*-Value ^1^
		Controlled (*n* = 124)	Uncontrolled (*n* = 327)		Controlled (*n* = 154)	Uncontrolled (*n* = 297)	
Age, years	65 [59–72]	67 [62–73]	67 [58–72]	0.035	68 [61–74]	65 [59–72]	0.024
Female gender	269 (60)	71 (57)	198 (61)	0.50	98 (64)	171 (58)	0.2
Race				0.006			0.3
White	244 (54)	66 (53)	178 (54)		91 (59)	153 (52)	
Hispanic or Latino	105 (23)	40 (32)	65 (20)		31 (20)	74 (25)	
Black or African	99 (22)	17 (14)	82 (25)		32 (21)	67 (23)	
Asian	3 (0.7)	1 (1)	2 (1)		0 (0)	3 (1)	
BMI, kg/m^2^	30 [26–33]	28 [25–32]	30 [26–34]	0.001	30 [26–33]	30 [26–33]	0.2
25–29.9 kg/m^2^	157 (35)	47 (38)	110 (34)		52 (34)	105 (35)	
≥30 kg/m^2^	217 (48)	46 (37)	144 (52)		71 (46)	146 (49)	
Waist size, cm	101 [92–110]	98 [89–107]	102 [93–112]	<0.001	98 [92–109]	102 [93–111]	0.043
Smoking habit				0.3			0.6
Smoker	31 (7)	12 (10)	19 (6)		13 (8)	18 (6)	
Non-smoker	265 (59)	70 (56)	195 (60)		88 (57)	177 (60)	
Former smoker	155 (34)	42 (34)	113 (35)		53 (34)	102 (34)	
Time since smoking cessation, years	20 [11–30]	21 [12–32]	20 [10–30]	0.6	20 [10–29]	21 [10–32]	0.4
Marital status				0.1			0.4
Single	68 (15)	11 (9)	57 (17)		24 (16)	44 (15)	
Married or in a relationship	240 (53)	74 (6)	166 (51)		77 (50)	163 (55)	
Divorced	63 (14)	19 (15)	44 (13)		27 (18)	36 (12)	
Widowed	80 (18)	20 (16)	60 (18)		26 (17)	54 (18)	
Educational level	n = 450		n = 326	--		n = 296	--
Low education *	356 (79)	93 (75)	263 (81)		112 (73)	244 (82)	
Medium education †	64 (14)	18 (15)	46 (14)		25 (16)	39 (13)	
High education ‡	30 (7)	13 (10)	17 (5)		17 (11)	13 (5)	
Currently employed	103 (23)	29 (23)	74 (23)	0.9	38 (25)	65 (22)	0.5
Self-reported low income	260 (58)	61 (49)	199 (61)	0.025	82 (53)	178 (60)	0.2
Sedentary lifestyle	200 (44)	56 (45)	144 (44)	0.8	57 (37)	143 (48)	0.024
PHQ-9 score	6 [2–11]	6 [3–10]	6 [2–11]	0.7	6 [2–10]	7 [3–12]	0.036
Positive depression screen (PHQ-9 ≥ 10)	138 (31)	39 (31)	99 (30)	0.8	37 (24)	101 (34)	0.029

Caption: The data are *n* (%) and median [IQR]. Abbreviations: BMI, body mass index; cm, centimeters; kg, kilograms; m, meters; SD, standard deviation; T2D, type 2 diabetes. ^1^ the Wilcoxon rank sum test for continuous variables; Pearson’s Chi-squared or Fisher’s exact tests for categorical variables. * less than primary, primary, and lower secondary. † upper secondary and post-secondary non-tertiary. ‡ short-cycle tertiary, bachelor’s or equivalent, master’s, and doctoral education.

**Table 3:** The comorbidities and CV risk of the study population.

	Overall (*n* = 451)	Hypertension (*n* = 451)		*p*-Value ^1^	T2D (*n* = 451)		*p*-Value ^1^
		Controlled (*n* = 124)	Uncontrolled (*n* = 327)		Controlled (*n* = 154)	Uncontrolled (*n* = 297)	
Additional comorbidities/Target Organ Damage							
Dyslipidemia	395 (88)	115 (93)	280 (86)	0.041	140 (91)	255 (86)	0.12
Clinical ASCVD	197 (44)	68 (55)	129 (39)	0.003	68 (44)	129 (43)	0.9
Acute coronary syndromes	30 (7)	13 (10)	17 (5)	0.044	10 (7)	20 (7)	>0.9
Chronic coronary syndrome	105 (23)	34 (27)	72 (22)	0.2	35 (23)	70 (24)	0.8
Myocardial infarction	85 (19)	31 (25)	54 (17)	0.040	24 (16)	61 (21)	0.2
Angina pectoris	30 (7)	12 (10)	18 (6)	0.11	10 (7)	20 (7)	>0.9
Stroke	36 (8)	10 (8)	26 (8)	>0.9	12 (8)	24 (8)	>0.9
Transient ischemic attack	14 (3)	3 (2)	11 (3)	0.8	6 (4)	8 (3)	0.6
Coronary or another arterial revascularization	63 (14)	27 (22)	36 (11)	0.003	18 (12)	45 (15)	0.3
Microvascular complications	177 (39)	46 (37)	131 (40)	0.6	46 (30)	131 (44)	0.03
Retinopathy	54 (12)	15 (12)	39 (12)	>0.9	10 (7)	44 (15)	0.01
Neuropathy	51 (11)	11 (9)	40 (12)	0.3	12 (8)	39 (13)	0.09
T2D-derived amputation	8 (2)	2 (2)	6 (2)	>0.9	1 (1)	7 (2)	0.3
Chronic Kidney Disease/nephropathy	120 (27)	35 (28)	85 (26)	0.6	37 (24)	83 (28)	0.4
Chronic kidney disease stage				<0.001			0.8
Moderate	100 (83)	24 (67)	76 (90)		32 (82)	68 (84)	
Severe	20 (17)	12 (33)	8 (10)		7 (18)	13 (16)	
Self-reported sleep disorders	60 (13)	20 (16)	40 (12)	0.3	27 (18)	33 (11)	0.057
Left ventricular hypertrophy	140 (31)	36 (29)	104 (32)	0.6	58 (38)	82 (28)	0.029
Abdominal obesity	299 (66)	67 (54)	232 (71)	<0.001	101 (66)	198 (67)	0.8
**CV risk according to the guideline**							
SCORE2 and SCORE2-OP	19 [12–30]	16 [11–23]	20 [13–32]	<0.001	19 [12–29]	20 [13–30]	0.7
Low-to-moderate	0 (0.0)	--	--		--	--	
High	100 (22%; 95% CI 18–26%)	--	--		--	--	
Very high	351 (78%; 95% CI 74–82%)	--	--		--	--	

Caption: The data are *n* (%) and median [IQR]. Abbreviations: ASCVD, atherosclerotic cardiovascular disease; T2D, type 2 diabetes. ^1^ the Wilcoxon rank sum test for continuous variables; Pearson’s Chi-squared or Fisher’s exact tests for categorical variables.

## Data Availability

The data underlying this article are available in the article and in its online Supplementary Material. The additional data underlying this article will be shared on reasonable request to the corresponding author.

## Supplementary Materials

The following supporting information can be downloaded at https://www.mdpi.com/article/10.3390/jcm15083022/s1, Methods S1. STROBE main checklist; Methods S2. Health Care Centers Participating in the Study; Methods S3. Patient-Reported Outcomes Questionnaires; Methods S4. Electronic Case Report Form; Methods S5. Variable and Outcome Definitions; Supplementary Table S1. Hemodynamic Profile and Antihypertensive Treatment Patterns; Supplementary Table S2. Glycemic Profile and Antidiabetic Treatment Patterns; Supplementary Table S3. Lipid Profile and Lipid-Lowering Treatment Patterns; Supplementary Table S4. Physician-Perceived vs. Guideline-Defined Control; Supplementary Table S5. Diagnostic Accuracy of Physician Perception; Supplementary Table S6. Multivariable Logistic Regression.

## Abbreviations

BP	Blood pressure
CV	Cardiovascular
HbA1c	Hemoglobin A1c
IQR	Interquartile range
LDL-C	Low-density lipoprotein cholesterol
LR	Likelihood ratio
T2D	Type 2 diabetes
