# Supplementary material for: Gaps Between Guidelines and Practice in Patients with Hypertension and Type 2 Diabetes: A Nationwide Cross-Sectional Study (SNAPSHOT–Brazil Study)
Source: J Clin Med. 2026 Apr 15;15(8):3022. doi: 10.3390/jcm15083022 (PMC13117057; doi:10.3390/jcm15083022)
Supplement: Supplementary file 1 [file jcm-15-03022-s001.zip › jcm-4215093-supplementary.pdf]

SUPPLEMENTARY MATERIAL

Gaps Between Guidelines and Practice in Patients with Hypertension and Type 2 Diabetes: A Nationwide Cross-Sectional Study (SNAPSHOT–Brazil Study)

Fernanda Marciano Consolim Colombo, MD[1]; Dalton Bertolim Précoma, MD, MSc, PhD[2]; Fábio Eduardo Camazzola, MD, MSc[3]; Eduardo Abib Junior, MD, MSc, PhD[4]; Denise Reis Franco, MD, MSc[5]; Lucelia Batista Neves Cunha Magalhaes, MD, MSc[6]; Antônio Carlos de Souza Spinelli, MD[7]; João Roberto Gemelli, MD[8]; Joao Lindolfo Cunha Borges, MD[9]; Renan Magalhães Montenegro Junior, MD, PhD[10]; Paulo Magno Martins Dourado, MD, PhD[11]; Renata Vital do Nascimento Lima, MD[12]; Mayara Lídia da Silva[12]; Douglas Mesadri Gewehr, MD[13]; Alleh Nogueira, MD[14]; Estefane Theophilo de Freitas Pereira[12]; Emilton Lima Junior, MD, PhD[13].

[1]*Hypertension Unit, Heart Institute of Medical School, University of São Paulo, Brazil*
[2]*Sociedade Hospitalar Angelina Caron, Campina Grande do Sul, Brazil*
[3]*Instituto de Pesquisas em Saúde (IPS), Fundação Universidade de Caxias do Sul (FUCS), Caxias do Sul, Brazil*
[4]*Scentryphar Pesquisa Clínica, Campinas, Brazil*
[5]*CPclin-Centro de Pesquisas Clínicas, São Paulo, Brazil*
[6]*Faculdade Zarns, Salvador, Brazil*
[7]*Cardiocentro Natal, Natal, Brazil*
[8]*Clínica Gemelli, Brasília, Brazil*
[9]*Centro de Pesquisa Clínica do Brasil, Brasília, Brazil*
[10]*Walter Cantidio University Hospital, Federal University of Ceará/Ebserh, Fortaleza, Brazil*
[11]*Pró Coração Cardiologia Preventiva, São Paulo, Brazil*
[12]*Medical Affairs Department, Servier Brazil Laboratories, Rio de Janeiro, Brazil*
[13]*Federal University of Paraná, Curitiba, Brazil*
[14]*Federal University of Rio Grande do Sul, Porto Alegre, Brazil*

Table of Contents

Methods S1. STROBE main checklist 3
Methods S2. Health Care Centers Participating in the Study 5
Methods S3. Patient-Reported Outcomes Questionnaires 6
Methods S3.1. Social Questionnaire 6
Methods S3.2. Patient Health Questionnaire-9 7
Methods S3.3. Hill-Bone Medication Adherence Scale 8
Methods S4. Electronic Case Report Form 9
Methods S5. Variable and Outcome Definitions 13
Methods S5.1. Patient-Reported Outcomes Questionnaire 13
Methods S5.2. Hypertension 13
Methods S5.3. Type 2 Diabetes (T2D) 14
Methods S5.4. Dyslipidaemia – LDL-cholesterol 14
Methods S5.5. Cardiovascular Risk 14
Supplementary Table S1. Hemodynamic Profile and Antihypertensive Treatment Patterns 17
Supplementary Table S2. Glycemic Profile and Antidiabetic Treatment Patterns 19
Supplementary Table S3. Lipid Profile and Lipid-Lowering Treatment Patterns 21
Supplementary Table S4. Physician-Perceived vs Guideline-Defined Control 23
Supplementary Table S5. Diagnostic Accuracy of Physician Perception 24
Supplementary Table S6. Multivariable Logistic Regression 25
Supplementary Table S6.1. Hypertension control 25
Supplementary Table S6.2. Type 2 Diabetes Control 26
Supplementary Table S6.3. LDL-C Control 27

Methods S1. STROBE main checklist

Topic	Item No	Checklist Item	Reported on
**Title and abstract**	1	(*a*) Indicate the study's design with a commonly used term in the title or the abstract	Manuscrit pg. 1
		(*b*) Provide in the abstract an informative and balanced summary of what was done and what was found	Manuscrit pg. 4
**Introduction**			
Background/rationale	2	Explain the scientific background and rationale for the investigation being reported	Manuscrit pg. 5-6
Objectives	3	State specific objectives, including any prespecified hypotheses	Manuscrit pg. 6
**Methods**			
Study design	4	Present key elements of study design early in the paper	Manuscrit pg. 6
Setting	5	Describe the setting, locations, and relevant dates, including periods of recruitment, exposure, follow-up, and data collection	Manuscrit pg. 6-7; Methods S2 (pg. 5)
Participants	6	(*a*) Give the eligibility criteria, and the sources and methods of selection of participants	Manuscrit pg. 7
Variables	7	Clearly define all outcomes, exposures, predictors, potential confounders, and effect modifiers. Give diagnostic criteria, if applicable	Manuscrit pg. 8-9<br>Methods S5 (pg. 13-16)
Data sources/ measurement	8*	For each variable of interest, give sources of data and details of methods of assessment (measurement). Describe comparability of assessment methods if there is more than one group	Manuscrit pg. 7-8;<br>Methods S3 (pg. 6-8) and S4 (pg. 9-12)
Bias	9	Describe any efforts to address potential sources of bias	Manuscrit pg. 10-11
Study size	10	Explain how the study size was arrived at	Manuscrit pg. 10
Quantitative variables	11	Explain how quantitative variables were handled in the analyses. If applicable, describe which groupings were chosen and why	Manuscrit pg. 7-8
		(*a*) Describe all statistical methods, including those used to control for confounding	Manuscrit pg. 10-11
		(*b*) Describe any methods used to examine subgroups and interactions	Manuscrit pg. 10-11
Statistical methods	12	(*c*) Explain how missing data were addressed	Manuscrit pg. 10-11
		(*d*) If applicable, describe analytical methods taking account of sampling strategy	Manuscrit pg. 10-11
		(*e*) Describe any sensitivity analyses	Manuscrit pg. 10-11

**Results**			
		(a) Report numbers of individuals at each stage of study—eg numbers potentially eligible, examined for eligibility, confirmed eligible, included in the study, completing follow-up, and analysed	Manuscrit pg. 11
Participants	13*	(b) Give reasons for non-participation at each stage	NA
		(c) Consider use of a flow diagram	NA
		(a) Give characteristics of study participants (eg demographic, clinical, social) and information on exposures and potential confounders	Manuscrit pg. 11-14;<br>Table S1 (pg. 17-18), S2 (pg. 19-20), and S3 (pg. 21-22)
Descriptive data	14*	(b) Indicate number of participants with missing data for each variable of interest	NA
Outcome data	15*	Report numbers of outcome events or summary measures	Manuscrit pg. 14-16; Table S4 (pg. 23) and S5 (pg. 24)
		(*a*) Give unadjusted estimates and, if applicable, confounder-adjusted estimates and their precision (eg, 95% confidence interval). Make clear which confounders were adjusted for and why they were included	Manuscrit pg. 14-16; Table S5 (pg. 25-27)
Main results	16	(*b*) Report category boundaries when continuous variables were categorized	Manuscrit pg. 14-16
		(*c*) If relevant, consider translating estimates of relative risk into absolute risk for a meaningful time period	NA
Other analyses	17	Report other analyses done—eg analyses of subgroups and interactions, and sensitivity analyses	NA
**Discussion**			
Key results	18	Summarise key results with reference to study objectives	Manuscrit pg. 16
Limitations	19	Discuss limitations of the study, taking into account sources of potential bias or imprecision. Discuss both direction and magnitude of any potential bias	Manuscrit pg. 21
Interpretation	20	Give a cautious overall interpretation of results considering objectives, limitations, multiplicity of analyses, results from similar studies, and other relevant evidence	Manuscrit pg. 21
Generalisability	21	Discuss the generalisability (external validity) of the study results	Manuscrit pg. 21
**Other information**			
Funding	22	Give the source of funding and the role of the funders for the present study and, if applicable, for the original study on which the present article is based	Manuscrit pg. 2

Abbreviations: MS, manuscript; suppl., supplement.

Methods S2. Health Care Centers Participating in the Study

The eleven healthcare centers in Brazil that participated in this study, along with their respective principal investigators (PIs) and the number of patients enrolled, are presented below:

Institution	PIs	Patients Enrolled
Heart Institute (InCor), Hospital das Clínicas of the University of São Paulo Medical School (HCFMPUSP), São Paulo, Brazil	Dr. Fernanda Consolim Colombo	124
Instituto Mantenedor de Ensino Superior da Bahia (IMES), Salvador, Brazil	Dr. Lucelia Batista N. C. Magalhães Magalhães	52
CPclin-Centro de Pesquisas Clínicas, São Paulo, Brazil	Dr. Denise Reis Franco	50
Clínica Gemelli, Brasília, Brazil	Dr. João Roberto Gemelli	40
Cardiocentro Natal, Natal, Brazil	Dr. Antonio Carlos de Souza Spinelli	40
Instituto de Pesquisas em Saúde (IPS), Fundação Universidade de Caxias do Sul (FUCS), Caxias do Sul, Brazil	Dr. Fabio Eduardo Camazzola	40
Hospital Universitário Walter Cantídio, Universidade Federal do Ceará, Fortaleza, Brazil	Dr. Renan Magalhães Montenegro Junior	32
Sociedade Hospitalar Angelina Caron, Campina Grande do Sul, Brazil	Dr. Dalton Bertolim Precoma	27
Centro de Pesquisa Clínica do Brasil, Brasília, Brazil	Dr. João Lindolfo Borges	19
Pró Coração Cardiologia Preventiva, São Paulo, Brazil	Dr. Paulo Magno Martins Dourado	14
Scentryphar Pesquisa Clínica, Campinas, Brazil	Dr. Eduardo Abib Jr	13

Methods S3. Patient-Reported Outcomes Questionnaires

Methods S3.1. Social Questionnaire

Education: What is your highest educational degree obtained?

- ☐ (0) Early childhood education (attainment: less than primary education)
- ☐ (1) Primary education
- ☐ (2) Lower secondary education
- ☐ (3) Upper secondary education
- ☐ (4) Post-secondary non-tertiary education
- ☐ (5) Short-cycle tertiary education
- ☐ (6) Bachelor's level education and equivalent
- ☐ (7) Master's level education and equivalent
- ☐ (8) Doctoral-level education

Marital status: What is your marital status?

- ☐ Married / in a relationship
- ☐ Single
- ☐ Divorced
- ☐ Widower

Socio-professional category?

- ☐ 1 Higher managerial, administrative, and professional occupations
  - ☐ 1.1 Large employers and higher managerial and administrative occupations
  - ☐ 1.2 Higher professional occupations
- ☐ 2 Lower managerial, administrative, and professional occupations
- ☐ 3 Intermediate occupations
- ☐ 4 Small employers and own account workers
- ☐ 5 Lower supervisory and technical occupations
- ☐ 6 Semi-routine occupations
- ☐ 7 Routine occupations
- ☐ 8 Never worked and long-term unemployed

Working status

Active

- ☐ Full-time worker
- ☐ Part-time worker

Non-active

- ☐ Student
- ☐ Retired
- ☐ Other

Physical Activity

- ☐ Sedentary
- ☐ Light
- ☐ Moderate
- ☐ Vigorous

> **Physical activity level scale:**
> **Sedentary:** refers to time spent sitting or lying down (except when sleeping), with very little energy expenditure. Examples of sedentary activities include: Sitting at work, Watching TV, Reading, Sewing, Computer use for non-active games or social networking, Sitting in a car, train, bus or tram.
> **Light:** activities are those that require standing up and moving around, either in the home, workplace or community. Some examples include: Housework like hanging out the washing, ironing and dusting or working at a standing workstation.
> **Moderate:** activities require some effort, but you can still talk while doing them (at least 4 hours / week). Examples of moderate intensity activities include: Brisk walking, Recreational swimming, Social tennis, Cleaning the windows at home.
> **Vigorous:** activities lead to harder breathing or puffing and panting (depending on your fitness) at least 8 hours / week. Examples of vigorous intensity activities include: Aerobics, Jogging, many competitive sports, Lifting, carrying and digging.
> *If activity varied a lot, please give an average covering the previous 3 months*
> ***Ref:*** *Australian Government – Health department - Levels of physical activity intensity*

Incomes

- ☐ Low (from xxxx to xxxx)
- ☐ Medium (from xxxx to xxxx)
- ☐ High (from xxxx to xxxx)

Methods S3.2. Patient Health Questionnaire-9

Over the last 2 weeks, how often have you been bothered

(Use "✔" to indicate your answer)

		Not at all	Several days	More than half the days	Nearly every day
1.	Little interest or pleasure in doing things	0	1	2	3
2.	Feeling down, depressed, or hopeless	0	1	2	3
3.	Trouble falling or staying asleep, or sleeping too much	0	1	2	3
4.	Feeling tired or having little energy	0	1	2	3
5.	Poor appetite or overeating	0	1	2	3
6.	Feeling bad about yourself — or that you are a failure or have let yourself or your family down	0	1	2	3
7.	Trouble concentrating on things, such as reading the newspaper or watching television	0	1	2	3
8.	Moving or speaking so slowly that other people could have noticed? Or the opposite — being so fidgety or restless that you have been moving around a lot more than usual	0	1	2	3
9.	Thoughts that you would be better off dead or of hurting yourself in some way	0	1	2	3

For Office coding: .... 0 .... + ... + ... + ...
=Total Score: ______

If you checked off any problems, how difficult have these problems made it for you to do your work, take care of things at home, or get along with other people?			
Not difficult at all	Somewhat difficult	Very difficult	Extremely difficult
☐	☐	☐	☐

Methods S3.3. Hill-Bone Medication Adherence Scale

For each question below, please answer for the disease you are suffering from: Hypertension, Hypercholesterolemia, and Type 2 Diabetes in the dedicated column.

Use "✔" to indicate your answer.

		Medical Condition		
		For Hypertension	For Hypercholesterolemia	For Type 2 Diabetes
1.	How often do you forget to take your Medical Conditions medicine?	1. All of the Time<br>2. Most of the Time<br>3. Some of the Time<br>4. None of the Time	1. All of the Time<br>2. Most of the Time<br>3. Some of the Time<br>4. None of the Time	1. All of the Time<br>2. Most of the Time<br>3. Some of the Time<br>4. None of the Time
2.	How often do you forget to take your Medical Conditions medicine?	1. All of the Time<br>2. Most of the Time<br>3. Some of the Time<br>4. None of the Time	1. All of the Time<br>2. Most of the Time<br>3. Some of the Time<br>4. None of the Time	1. All of the Time<br>2. Most of the Time<br>3. Some of the Time<br>4. None of the Time
3.	How often do you forget to get prescriptions filled?	1. All of the Time<br>2. Most of the Time<br>3. Some of the Time<br>4. None of the Time	1. All of the Time<br>2. Most of the Time<br>3. Some of the Time<br>4. None of the Time	1. All of the Time<br>2. Most of the Time<br>3. Some of the Time<br>4. None of the Time
4.	How often do you run out of Medical Conditions pills?	1. All of the Time<br>2. Most of the Time<br>3. Some of the Time<br>4. None of the Time	1. All of the Time<br>2. Most of the Time<br>3. Some of the Time<br>4. None of the Time	1. All of the Time<br>2. Most of the Time<br>3. Some of the Time<br>4. None of the Time
5.	How often do you skip your Medical Conditions medicine before you go to the doctor?	1. All of the Time<br>2. Most of the Time<br>3. Some of the Time<br>4. None of the Time	1. All of the Time<br>2. Most of the Time<br>3. Some of the Time<br>4. None of the Time	1. All of the Time<br>2. Most of the Time<br>3. Some of the Time<br>4. None of the Time
6.	How often do you miss taking your Medical Conditions pills when you feel better?	1. All of the Time<br>2. Most of the Time<br>3. Some of the Time<br>4. None of the Time	1. All of the Time<br>2. Most of the Time<br>3. Some of the Time<br>4. None of the Time	1. All of the Time<br>2. Most of the Time<br>3. Some of the Time<br>4. None of the Time
7.	How often do you miss taking your Medical Conditions pills when you feel sick?	1. All of the Time<br>2. Most of the Time<br>3. Some of the Time<br>4. None of the Time	1. All of the Time<br>2. Most of the Time<br>3. Some of the Time<br>4. None of the Time	1. All of the Time<br>2. Most of the Time<br>3. Some of the Time<br>4. None of the Time
8.	How often do you take someone else's Medical Conditions pills?	1. All of the Time<br>2. Most of the Time<br>3. Some of the Time<br>4. None of the Time	1. All of the Time<br>2. Most of the Time<br>3. Some of the Time<br>4. None of the Time	1. All of the Time<br>2. Most of the Time<br>3. Some of the Time<br>4. None of the Time
9.	How often do you miss taking your Medical Conditions pills when you are careless?	1. All of the Time<br>2. Most of the Time<br>3. Some of the Time<br>4. None of the Time	1. All of the Time<br>2. Most of the Time<br>3. Some of the Time<br>4. None of the Time	1. All of the Time<br>2. Most of the Time<br>3. Some of the Time<br>4. None of the Time

Methods S4. Electronic Case Report Form

Selection by the physician

Demography	
**Country**	....
Investigator firstname	....
Investigator name	....
Date of visit	I__I__I/I__I__I/I__I__I__I__I
Gender	○ Male ○ Female
Age	I__I__I__I years
Ethnicity	☐ Hispanic or Latino<br>☐ Not Hispanic or Latino
Race	☐ Mexican, Mexican American, Chicano/a<br>☐ Puerto Rican<br>☐ Cuban<br>☐ Anthoner Hispanic, latino/a or Spanish origin<br>☐ White<br>☐ Black or African<br>☐ American Indian or Alaska Native<br>☐ Asian<br>☐ Pacific Islander
Weight	I__I__I__I kg
BMI	I__I__I , I__I kg/m²
Height	I__I__I__I cm
Smoking habit	○ smoker ○ non smoker ○ former smoker
Number of cigarettes:	I__I__I__I cigarettes/day
Year of stop:	I__I__I__I__I

Cardiovascular Risk	
Cardiovascular Risk (according to your judgment)	○ Low<br>○ Moderate<br>○ High<br>○ Very high
Additional comorbidities	☐ Acute Coronary syndromes<br>☐ Myocardial infarction<br>☐ Angina<br>☐ Stroke<br>☐ Transient ischemic attack<br>☐ Coronary or other arterial revascularization<br>☐ Peripheral arterial disease (presumed to be of atherosclerotic origin)
Hypertensive Left Ventricular Hypertrophy	○ Yes ○ No
Diabetes mellitus	○ Yes ○ No
Type of diabetes	○ Type I ○ Type II
Target organ damage	○ Yes ○ No
Chronic Kidney Disease	○ Yes ○ No
Severity	○ Moderate ○ Severe

Hypertension	
Hypertension	◯ Yes ◯ No
Year of diagnosis	|__|__|__|__|
Current SBP	|__|__|__| mmHg
Current DBP	|__|__|__| mmHg
Resting heart rate	|__|__|__| beats/min
Hypertension treated	◯ Yes ◯ No
Drug class for the treatment of hypertension	☐ ACEIs<br>☐ ARBs<br>☐ CCBs<br>☐ Diuretics<br>☐ Beta blockers<br>☐ Central Antihypertensive drugs<br>☐ Other BP lowering drugs
If Other, specify	....
Use of Single Pill Combination for hypertension	◯ Yes ◯ No
Considered as controlled?	◯ Yes ◯ No

Protocole : BP AND HbA1c SNAPSHOT -- INT Centre |__|__|__| Patient : _ _ _ _ _ _ _

Dyslipidemia	
Dyslipidemia	◯ Yes ◯ No
Year of diagnosis	|__|__|__|__|
Date (Last assessment)	|__|__|/|__|__|/|__|__|__|__|
Please provide values for the following parameters from this last assessment :	
Total Cholesterol	
HDLc	
LDLc	
Triglycerides	
Dyslipidemia treated?	◯ Yes ◯ No
Dietary supplements for the treatment of dyslipidaemias	◯ Yes ◯ No
Drug class for the treatment of dyslipidaemias	☐ Statins<br>☐ Cholesterol absorption inhibitor<br>☐ PCSK9 inhibitors<br>☐ Fibrates<br>☐ Other lipid lowering drugs
If Statin, intensity of statin treatment	◯ Mild ◯ Moderate ◯ High
If Other, specify	....
Use of Single Pill Combination for dyslipidemia	◯ Yes ◯ No
Use of Single Pill Combination for hypertension and dyslipidemia	◯ Yes ◯ No
Total number of daily pills (all treatment)	|__|__| pills
Considered as controlled?	◯ Yes ◯ No

Type 2 diabetes	
Type 2 diabetes	○ Yes ○ No
Year of diagnosis	|__|__|__|__|
Parameters (last assessment available) :	☐ 1 - Glycated hemoglobin HbA1c<br>☐ 2 - Fasting glucose
1- Glycated hemoglobin HbA1c	☐ 4% = 20 mmol/mol<br>☐ 4.5% = 26 mmol/mol<br>☐ 5% = 31 mmol/mol<br>☐ 5.5% = 37 mmol/mol<br>☐ 6% = 42 mmol/mol<br>☐ 6.5% = 48 mmol/mol<br>☐ 7% = 53 mmol/mol<br>☐ 7.5% = 58 mmol/mol<br>☐ 8% = 64 mmol/mol<br>☐ 8.5% = 69 mmol/mol<br>☐ 9% = 75 mmol/mol<br>☐ 9.5% = 80 mmol/mol<br>☐ 10% = 86 mmol/mol<br>☐ 10.5% = 91 mmol/mol<br>☐ 11% = 97 mmol/mol<br>☐ 11.5% = 102 mmol/mol<br>☐ 12% = 108 mmol/mol<br>☐ 12.5% = 113 mmol/mol<br>☐ 13% = 119 mmol/mol<br>☐ 13.5% = 124 mmol/mol<br>☐ 14% = 130 mmol/mol<br>☐ 14.5% = 135 mmol/mol<br>☐ 15% = 140 mmol/mol<br>☐ 15.5% = 146 mmol/mol<br>☐ 16% = 151 mmol/mol
date of assessment	|__|__|/|__|__|/|__|__|__|__|
2- Fasting glucose	
date of assessment	|__|__|/|__|__|/|__|__|__|__|
Type 2 diabetes related complications	○ Yes ○ No
Microvascular complications	○ Yes ○ No
please specify:	☐ Retinopathy ☐ Nephropathy ☐ Neuropathy

Macrovascular complications: Atherosclerotic cardiovascular disease (ASCVD)	○ Yes ○ No
please specify:	☐ Acute Coronary syndromes<br>☐ Myocardial infarction<br>☐ Angina<br>☐ Stroke<br>☐ Transient ischemic attack<br>☐ Coronary or other arterial revascularization<br>☐ Peripheral arterial disease (presumed to be of atherosclerotic origin)
Type 2 diabetes treated	○ Yes ○ No
Treatment class	☐ Biguanide (metformin)<br>☐ Alpha-glucosidase inhibitor (acarbose, miglitol)<br>☐ Sulfonylurea<br>☐ Glinide (repaglinide, nateglinide)<br>☐ dipeptidyl peptidase-4 inhibitor (DPP4i)<br>☐ Sodium-glucose co-transporter 2 inhibitor (SGLT2i)<br>☐ glucagon-like peptide-1 analogue (GLP-1 RA)<br>☐ Insulin
Sulfonylurea - please specify:	○ First generation (tolbutamide, chlorpropamide)<br>○ Second Generation (glibenclamide, gliclazide, glipizide)<br>○ Third Generation (gliclazide MR, glimepiride, glipizide XL)
GLP-1 RA - please specify:	○ Injectable ○ Oral
Insulin - please specify:	☐ Short acting ☐ Long acting
Use of antidiabetic Single Pill Combination	○ Yes ○ No

Methods S5. Variable and Outcome Definitions

Methods S5.1. Patient-Reported Outcomes Questionnaire

1. According to social factors:
   - Education: primary (early childhood education or primary education), medium (lower secondary, upper secondary, or post-secondary non-tertiary), high (short cycle tertiary, bachelor level, master level, or doctorate);
   - Marital status: married/in a relationship, single, divorced, widower;
   - Working status: active, non-active Socio-professional categories: managerial (higher managerial or lower managerial), non-managerial (intermediate or small employers or lower supervisory or semi-routine or routine), never worked/long-term unemployed;
   - Incomes: low, medium, high;
   - Physical activities: sedentary, light, moderate, vigorous.

Methods S5.2. Hypertension

1. Time since diagnosis (in years) was derived as: (year of visit date – year of diagnosis).
2. Hypertension was classified as follows:
   - Optimal: SBP <120 and DBP <80;
   - Normal: SBP [120-129] and/or DBP [80-84];
   - High normal: SBP [130-139] and/or DBP [85-89];
   - Grade 1: SBP [140-159] and/or DBP [90-99];
   - Grade 2: SBP [160-179] and/or DBP [100-109];
   - Grade 3: SBP ≥180 and/or DBP ≥110;
   - Isolated systolic hypertension: SBP ≥140 and DBP <90.

   The highest grade will be used in case of discordance between SBP and DBP.

3. Control status, according to guidelines, for hypertension (<u>controlled</u> vs. <u>not controlled</u>) was derived as follows:
   - In non-treated patients (not under antihypertensive medication):
     - For patients aged <80 years old, BP will be considered as controlled if SBP <140 mmHg and DBP <90 mmHg.
     - For patients aged ≥80 years old, BP control is defined as SBP <160 mmHg and DBP <90 mmHg.
   - In treated patients:

- For patients aged <65 years old without chronic kidney disease, BP control is defined as SBP<130 mmHg and DBP<80 mmHg.
- For patients aged <65 years old with chronic kidney disease and patients aged ≥65 years old, BP control is defined as SBP<140 mmHg and DBP<80 mmHg.

Methods S5.3. Type 2 Diabetes (T2D)

1. Time since T2D diagnosis (in years) was derived as: (year of visit date – year of diagnosis).
2. HbA1c was described by category: <6.5%, [6.5-6.9]%, [7.0-7.9]%, [8.0-8.9]%, and ≥9.0%.
3. Control status, according to guidelines, for T2D (controlled vs. not controlled) was considered as controlled if HbA1c <7% (<53 mmol/mol).
4. Among patients with micro/macro vascular complications, patients with multiple micro/macro vascular complications were identified as:
   - Yes: more than one micro/macro vascular complication;
   - No: Only one micro/macro vascular complication.

Methods S5.4. Dyslipidaemia – LDL-cholesterol

1. Time since diagnosis (in years) will be derived as: (year of visit date – year of diagnosis).
2. Control status, according to guidelines, for LDL-cholesterol (controlled vs. not controlled) was derived in accordance with the SCORE2 and SCORE2-OP, as follows:
   - For patients with very high cardiovascular risk (calculated), dyslipidemia control is defined as LDLc <1.4 mmol/L (<55 mg/dL);
   - For patients with high cardiovascular risk (calculated), dyslipidemia control is defined as LDLc <1.8 mmol/L (<70 mg/dL);
   - For patients with low-to-moderate cardiovascular risk (calculated), dyslipidemia control is defined as LDLc <2.6 mmol/L (<100 mg/dL).

Methods S5.5. Cardiovascular Risk

The calculated cardiovascular risk was derived using comorbidities and scoring charts SCORE 2 and SCORE2-OP of the European Association of Preventive Cardiology. For scoring, former smokers will be treated as non-smokers, regardless of the date of cessation.

ESC Reccomendations | SCORE2 and SCORE2-OP	
**Low-to-moderate**	• Age <50 years: score <2.5%;<br>• Age 50-69 years: score <5%;

	• Age ≥70 years: score <7.5%;<br>• Patients with type 2 diabetes without target organ damage, time since diagnosis <10 years and without additional CV risk factors.
**High**	• Moderate chronic kidney disease;<br>• Patients with type 2 diabetes, without target organ damage, time since diagnosis <10 years and with additional CV risk factors;<br>• Patients with type 2 diabetes without target organ damage and time since diagnosis ≥10 years;<br>• Age <50 years: score [2.5-7.5[%;<br>• Age 50-69 years: score [5-10[%;<br>• Age ≥70 years: score [7.5-15[%.
**Very High-risk**	• Severe chronic kidney disease;<br>• Diabetes mellitus with target organ damage;<br>• Diabetes mellitus with microvascular complications;<br>• Presence of at least one additional CV comorbidity (acute coronary syndrome, myocardial infarction, angina, stroke, transient ischemic attack, coronary or other arterial revascularization or peripheral artery disease);<br>• Age <50 years: score ≥7.5%;<br>• Age 50-69 years: score ≥10%;<br>• Age ≥70 years: score ≥15%.
If a patient had items from different levels of score, we used the highest level, i.e. a patient with type 2 diabetes without target organ damage and time since diagnosis ≥10 years and with severe chronic kidney disease, score was 'Very high'.	

The concordance between both scores (calculated vs the investigator's estimation) was described as follows:

SCORE2 and SCORE2-OP

- **Accurate:** a match between the CV risk assessed by the investigator and the calculated CV risk. The CV risk assessment is also considered accurate if the investigator assessed the CV risk as low or moderate, and the computed CV risk is low-to-moderate
- **Overestimated by the investigator:**
  - Considered as high by the investigator, but the calculated CV risk is low-to-moderate.
  - Considered as very high by the investigator, but the calculated CV risk is low to moderate or high.
- **Underestimated by the investigator:**
  - Considered as low to moderate by the investigator, but the calculated CV risk is high or very high.
  - Considered high by the investigator, but the calculated CV risk is very high.

		**Calculated CV risk – SCORE2 and SCORE2-OP**		
		**Low-to-moderate**	**High**	**Very high**
**CV risk**	**Low**	Accurate	underestimated	underestimated

**assessed by the investigator**	**Moderate**	Accurate	underestimated	underestimated
	**High**	overestimated	Accurate	underestimated
	**Very high**	overestimated	overestimated	Accurate

Supplementary Table S1. Hemodynamic Profile and Antihypertensive Treatment Patterns

	Overall n=451	CV Risk		p-value	Control Status for Hypertension		p-value[1]
		High Risk n=100	Very High Risk n=351		Controlled n=124	Uncontrolled n=327	
**Hemodynamic evaluation**							
Time since diagnosis, years	16 [9-25]	13 [8-20]	18 [10-28]	<0.001	14 [7-23]	18 [10-27]	0.03
Current SBP, mm Hg	140 [126-158]	133 [122-143]	140 [127-160]	0.001	120 [110-129]	148 [134-166]	<0.001
Current DBP, mm Hg	80 [70-90]	84 [73-91]	80 [70-90]	0.055	70 [64-72]	85 [80-93]	<0.001
Hypertension stages, mmHg							
<120/80	53 (12)	11 (11)	42 (12)		53 (43)	0 (0)	
120–129/80–84	143 (32)	35 (35)	108 (31)		42 (34)	101 (31)	
130–139/85–89	99 (22)	29 (29)	70 (20)		29 (23)	70 (21)	
140–159/90–99	101 (22)	16 (16)	85 (24)		0 (0)	101 (31)	
160–179/100–109	37 (8)	8 (8)	29 (8)		0 (0)	37 (11)	
≥180/110	18 (4)	1 (1)	17 (5)		0 (0)	18 (6)	
Isolated systolic hypertension	121 (27)	15 (15)	106 (30)		0 (0)	121 (37)	
Resting HR, beats/minute	72 [65-81]	76 [69-84]	72 [64-80]	0.005	70 [64-79]	74 [66-82]	0.038
**Treatment evaluation**							
Patients on therapy	443 (98)	97 (97)	346 (99)	0.4	122 (98)	321 (98)	>0.9
Adherence to treatment according to HB-MAS	35 [33-36]	35 [33-36]	35 [34-36]	0.6	36 [34-36]	35 [33-36]	0.092
Single-pill combination	72 (16)	21 (21)	51 (15)	0.12	35 (28)	37 (11)	<0.001
Daily pills for hypertension	4 [2-6]	3 [2-4]	4 [2-7]	<0.001	3 [2-5]	4 [2-7]	<0.001
Patients with at least one BP medication							
ACEI	179 (40)	32 (32)	147 (42)	0.075	47 (38)	132 (40)	0.6
ARB	231 (51)	60 (60)	171 (49)	0.046	61 (49)	170 (52)	0.6
CCB	221 (49)	36 (36)	185 (53)	0.003	47 (38)	174 (53)	0.004
Diuretics	268 (59)	60 (60)	208 (59)	0.9	72 (58)	196 (60)	0.7
BB	250 (55)	26 (26)	224 (64)	<0.001	71 (57)	179 (55)	0.6
Central antihypertensive medication	61 (14)	13 (13)	48 (14)	0.9	3 (2)	58 (18)	<0.001

Other	80 (18)	12 (12)	68 (19)	0.09	7 (6)	73 (22)	<0.001

Caption: Data are n (%), mean±SD, or median [IQR].

Abbreviations: ACEI, angiotensin-converting enzyme inhibitors; ARB, angiotensin II receptor blocker; BB, beta-blockers; CCB, calcium channel blockers; DBP, diastolic blood pressure; HB-MAS, Hill-Bone Medication Adherence Scale; HR, heart rate; mm Hg, millimeters of mercury; PHQ-9, Patient Health Questionnaire-9; SD, standard deviation; SBP, systolic blood pressure.

[1]Wilcoxon rank sum test for continuous variables; Pearson's Chi-squared or Fisher's exact tests for categorical variables.

Supplementary Table S2. Glycemic Profile and Antidiabetic Treatment Patterns

	Overall n=451	Cardiovascular Risk		p-value	Control Status for T2D		p-value[1]
		High Risk n=100	Very High Risk n=351		Controlled n=154	Uncontrolled n=297	
Glycemic evaluation							
Time since diagnosis, years	12 [5-20]	10 [3-15]	13 [6-22]	<0.001	9 [4-18]	13 [7-22]	<0.001
HbA1c, %	7.0 [6.5-8.0]	7.0 [6.0-8.5]	7.0 [6.5-8.0]	0.6	6.0 [6.0-6.5]	7.5 [7.0-9.0]	<0.001
HbA1c category				0.10			<0.001
<6.5%	109 (24)	29 (29)	80 (23)		109 (71)	0 (0)	
6.5–<7.0%	45 (10)	11 (11)	34 (10)		45 (29)	0 (0)	
7.0–<8.0%	154 (34)	25 (25)	129 (37)		0 (0)	154 (52)	
8.0–<9.0%	62 (14)	11 (11)	51 (15)		0 (0)	62 (21)	
≥9.0%	81 (18)	24 (24)	57 (16)		0 (0)	81 (27)	
Fasting glucose, mg/dL	129 [107-161]	129 [108-158]	129 [107-164]	0.5	107 [96-129]	140 [128-187]	<0.001
Treatment evaluation							
Patients on therapy	444 (98)	97 (97)	347 (99)	0.2	152 (99)	292 (98)	>0.9
Adherence to treatment according to HB-MAS	35 [33-36]	35 [33-36]	35 [34-36]	0.3	36 [34-36]	35 [33-36]	0.051
Single-pill combination	77 (17)	18 (18)	59 (17)	0.8	38 (25)	39 (13)	0.002
Daily pills for T2D	3 [2-4]	2 [2-4]	3 [2-4]	0.3	2 [1-3]	3 [2-4]	<0.001
Patients with at least one antidiabetic medication							
Metformin	374 (83)	152	134 (88.2)	0.2	134 (87)	240 (81)	0.10
Insulin	153 (34)	152	15 (9.9)	0.06	15 (10)	138 (46)	<0.001
SGLT2i	143 (32)	152	48 (31.6)	0.10	48 (31)	95 (32)	0.9
Sulfonylurea	134 (30)	152	33 (21.7)	0.06	33 (21)	101 (34)	0.006
DPP4i	49 (11)	152	20 (13.2)	>0.9	20 (13)	29 (10)	0.3
GLP-1 RA	22 (5)	152	10 (6.6)	0.2	10 (7)	12 (4)	0.3
Other*	7 (2)	152	5 (2.1)	--	6 (4)	1 (1)	--

Caption: Data are n (%) or median [IQR].

Abbreviations: dL, deciliters; DPP4i, Dipeptidyl peptidase 4 inhibitor; GLP-1, Glucagon-like peptide-1 receptor agonists; HbA1c, hemoglobin A1c; HB-MAS, Hill-Bone Medication Adherence Scale; HR, heart rate; mg, milligrams; PHQ-9, Patient Health Questionnaire-9; SD, standard deviation; SGLT2i, sodium-glucose transport protein 2 inhibitors; T2D, type 2 diabetes.

*Other medications for T2D included alpha-glucosidase inhibitors, glinides, or pioglitazone.
[1]Wilcoxon rank sum test for continuous variables; Pearson's Chi-squared or Fisher's exact tests for categorical variables.

Supplementary Table S3. Lipid Profile and Lipid-Lowering Treatment Patterns

	Overall n=451	Cardiovascular Risk		p-value	Control Status for LDL-C		p-value[1]
		High Risk n=100	Very High Risk n=351		Controlled n=59	Uncontrolled n=392	
Lipid profile evaluation							
Time since diagnosis, years	10 [5-16]	10 [4-13]	10 [6-18]	0.001	8 [4-14]	10 [5-17]	0.11
LDL-C, mg/dL	85 [71-103]	85 [77-98]	85 [71-104]	0.8	45 [37-52]	85 [83-107]	<0.001
LDL-C category				0.2			<0.001
<55 mg/dL	51 (11)	15 (15)	36 (10)		51 (86)	0 (0)	
55–<70 mg/dL	54 (12)	8 (8)	46 (13)		8 (14)	46 (12)	
70–<100 mg/dL	221 (49)	53 (53)	168 (48)		0 (0)	221 (56)	
≥100 mg/dL	125 (28)	24 (24)	101 (29)		0 (0)	125 (32)	
Total cholesterol, mg/dL	163 [143-165]	163 [143-165]	163 [143-165]	0.5	117 [102-128]	163 [156-192]	<0.001
HDL-C, mg/dL	43 [39-47]	43 [43-49]	43 [38-47]	0.081	39 [32-49]	43 [40-47]	0.012
Non-HDL-C, mg/dL	120 [98-139]	120 [95-134]	120 [98-139]	0.4	72 [64-92]	120 [112-143]	<0.001
Triglycerides, mg/dL	152 [123-195]	152 [122-176]	152 [125-196]	0.4	133 [93-207]	152 [132-192]	0.005
Treatment evaluation							
Patients on therapy	379 (84)	75 (100)	304 (87)	0.005	59 (100)	320 (82)	<0.001
Adherence to treatment according to HB-MAS	35 [34-36]	35 [34-36]	35 [34-36]	0.2	35 [34-36]	35 [34-36]	0.8
Single-pill combination	47 (10)	11 (11)	36 (10)	0.8	19 (32)	28 (7)	<0.001
Daily pills for dylipidemia	1 [1-1]	1 [1-1]	1 [1-1]	0.015	1 [1-1]	1 [1-1]	0.2
Patients with at least one lipid-lowering drug							
Statin use	376 (83)	75 (75)	301 (86)	0.011	59 (100)	317 (81)	<0.001
Statin intensity				<0.001			0.3
Low	94 (25)	31 (41)	63 (21)		18 (31)	76 (24)	
Moderate	125 (33)	27 (36)	98 (33)		22 (37)	103 (32)	
High	157 (42)	17 (23)	140 (47)		19 (32)	138 (44)	
Ezetimibe use	55 (12)	10 (10)	45 (13)	0.4	19 (32)	36 (9)	<0.001
PCSK9 inhibitor use	0 (0)	0 (0)	0 (0)	--	0 (0)	0 (0)	--

Fibrate	22 (5)	3 (3)	19 (5.4)	0.4	4 (7)	18 (5)	0.5
Other lipid-modifying therapy	1 (0.2)	0 (0)	1 (0.3)	>0.9	0 (0)	1 (0.3)	>0.9

Caption: Data are n (%) or median [IQR].

Abbreviations: dL, deciliters; HB-MAS, Hill-Bone Medication Adherence Scale; HR, heart rate; HDL-C, high-density lipoprotein cholesterol; LDL-C, low-density lipoprotein cholesterol; mg, milligrams; PCSK9, Proprotein convertase subtilisin/kexin type 9; PHQ-9, Patient Health Questionnaire-9; SD, standard deviation; T2D, type 2 diabetes.

[1]Wilcoxon rank sum test for continuous variables; Pearson’s Chi-squared or Fisher’s exact tests for categorical variables.

Supplementary Table S4. Physician-Perceived vs Guideline-Defined Control

Outcome	n	Guideline-controlled (95% CI)	Physician-controlled (95% CI)	Prevalence difference (95% CI)	p-value[1]	Acuracy of Physician-Perceived		
						Accurate	Overestimated	Underestimated
BP	451	27% (23-32%)	56% (51-61%)	29% (22-35%)	<0.001	70% (66-74%)	29% (25-34%)	1% (0-2%)
LDL-C	451	13% (10-17%)	47% (42%-52%)	34% (28-40%)	<0.001	63% (58-67%)	35% (31-40%)	2% (1-3%)
BP + LDL-C	451	6% (4-9%)	31% (26-35%)	25% (20-30%)	<0.001	75% (71-79%)	25% (21-29%)	0.2% (0.01-1.4%)
HbA1c	451	34% (30-39%)	--	--	--	--	--	--
BP + HbA1c	451	11% (8-14%)	--	--	--	--	--	--
LDL-C + HbA1c	451	6% (4-9%)	--	--	--	--	--	--
BP + LDL-c + HbA1c	451	3% (2-6%)	--	--	--	--	--	--
CV risk	451	--	--	--	--	49% (45-54%)	0.7% (0.2-2.1%)	50% (45-55%)

Caption: Data are % (95% Confidence Interval).

Abbreviations: BP, blood pressure; CI, confidence interval; HbA1c; Glycated hemoglobin; LDL-C, low-density lipoprotein cholesterol.

[1]McNemar test for paired proportions.

Supplementary Table S5. Diagnostic Accuracy of Physician Perception

Outcome	n	Sens (95% CI)	Spec (95% CI)	LR+ (95% CI)	LR- (95% CI)	PPV (95% CI)	NPV (95% CI)	AUC-ROC (95% CI)	A (95% CI)
BP	451	0.98 (0.93-0.99)	0.60 (0.54-0.65)	2.42 (2.11-2.77)	0.04 (0.01-0.12)	0.48 (0.42-0.54)	0.98 (0.96-1.00)	0.79 (0.76-0.82)	0.70 (0.66-0.74)
LDL-C	451	0.88 (0.77-0.95)	0.59 (0.54-0.64)	2.16 (1.86-2.51)	0.20 (0.10-0.40)	0.25 (0.19-0.31)	0.97 (0.94-0.99)	0.74 (0.69-0.78)	0.63 (0.58-0.67)
BP + LDL-C	451	0.96 (0.81-1.00)	0.74 (0.69-0.78)	3.65 (3.06-4.34)	0.05 (0.01-0.34)	0.19 (0.13-0.26)	1.00 (0.98-1.00)	0.85 (0.81-0.89)	0.75 (0.71-0.79)

Caption: Data are % (95% Confidence Interval).

Abbreviations: AUC-ROC, area under the receiver-operating characteristic curve; BP, blood pressure; LDL-C, low-density lipoprotein cholesterol; LR+, positive likelihood ratio; LR–, negative likelihood ratio; NPV, negative predictive value; PPV, positive predictive value; Sens, sensitivity; Spec, specificity.

Supplementary Table S6. Multivariable Logistic Regression

Supplementary Table S6.1. Hypertension control

Characteristic	OR[1]	95% CI[1]	p-value
Age, per year	1.03	1.00, 1.07	**0.034**
Female sex	1.27	0.75, 2.17	0.4
Low income	0.71	0.42, 1.20	0.2
Post-secondary education	3.21	1.32, 7.98	**0.010**
PHQ-9 score	1.01	0.97, 1.06	0.7
Abdominal obesity	0.52	0.31, 0.87	**0.013**
Sedentary lifestyle	1.36	0.82, 2.28	0.2
Current smoker	2.38	1.00, 5.56	**0.046**
Chronic kidney disease	1.24	0.70, 2.19	0.5
Very high cardiovascular risk	1.18	0.59, 2.40	0.6
HB-MAS adherence score	1.03	0.95, 1.13	0.5
Single-pill combination	1.97	1.05, 3.67	**0.033**
Daily pills, per additional pill	0.90	0.82, 0.98	**0.029**
Time since diagnosis, per 5 years	0.89	0.79, 1.00	**0.045**

[1] OR = Odds Ratio, CI = Confidence Interval

Supplementary Table S6.2. Type 2 Diabetes Control

Characteristic	OR[1]	95% CI[1]	p-value
Age, per year	1.03	1.00, 1.06	**0.026**
Female sex	1.08	0.63, 1.87	0.8
Low income	1.31	0.78, 2.21	0.3
Post-secondary education	5.07	1.92, 14.5	**0.001**
PHQ-9 score	0.98	0.94, 1.03	0.4
Abdominal obesity	1.15	0.68, 1.95	0.6
Sedentary lifestyle	0.60	0.36, 1.01	0.059
Current smoker	1.83	0.76, 4.33	0.2
ASCVD	1.03	0.62, 1.70	>0.9
Microvascular complications	0.58	0.34, 0.98	**0.045**
HB-MAS adherence score	1.11	1.02, 1.24	**0.028**
Single-pill combination	1.56	0.85, 2.88	0.2
Daily pills, per additional pill	0.67	0.56, 0.80	**<0.001**
Time since diagnosis, per 5 years	0.81	0.71, 0.92	**0.001**

[1] OR = Odds Ratio, CI = Confidence Interval

Supplementary Table S6.3. LDL-C Control

Characteristic	OR[1]	95% CI[1]	p-value
Age, per year	1.04	1.01, 1.06	**0.004**
Female sex	1.42	0.88, 2.29	0.2
Low income	0.96	0.61, 1.51	0.9
Post-secondary education	4.48	1.89, 11.3	**<0.001**
PHQ-9 score	0.96	0.92, 1.00	0.081
Abdominal obesity	1.00	0.62, 1.61	>0.9
Sedentary lifestyle	0.71	0.45, 1.12	0.14
Current smoker	1.51	0.66, 3.39	0.3
ASCVD	1.01	0.64, 1.58	>0.9
HB-MAS adherence score	1.05	0.97, 1.16	0.2
Single-pill combination	1.74	0.87, 3.45	0.11
Daily pills, per additional pill	0.63	0.35, 0.99	0.087
Time since diagnosis, per 5 years	0.97	0.85, 1.10	0.6

[1] OR = Odds Ratio, CI = Confidence Interval
